# Mode of Preventing Bed Sores

**Published:** 1848

**Authors:** 


ARTICLE IX.—*Simple mode of preventing Bed-sores.*

The following ingenious method of preventing those dreadful specimens of disease or neglect sometimes presented in even our best regulated institutions, is suggested by Dr. Purefoy, in a former number of the *Dublin Medical Press.* The Doctor may be allowed to describe his practice in his own words:

"Having lately to treat a case of compound fracture of the tibia in an old man, the leg was comfortably placed upon the double inclined plane, and the case went on very favorably for some days, when it was thought that the leg must be placed upon the side, in order to relieve intolerable pain of the heel, and to obviate sloughing of the integuments. In this dilemma, it occurred to me to try to support the heel upon a bladder partially inflated, since pads failed completely in affording the desired relief. An ox-bladder, previously moistened in tepid water, and afterwards oiled, was placed under the heel in a flaccid state, and subsequently filled gently with air, so as to give the heel the necessary elevation, and promote, as far as might be, the comfort of the patient. The experiment was successful beyond my most sangnine hope, as the air flowed underneath and upwards by the sides of the foot and ankle, thus affording an unusually agreeable and secure support to the foot and instep, at the same time relieving the heel from undue pressure: The old man exclaimed in rapture, 'O, sir, I'm in heaven!' Suffice it to say, that by renewing the bladder once only, the cure was perfected so far at the end of a month, that the patient could leave his bed, and during this time he was completely relieved from the intolerable pain which at first was so very troublesome. I have lately prevented the occurrence of bed-sores by the aid of a bladder placed under the buttocks, and rolled up in a soft napkin, having previously been partially filled with air, although the patient had been for nearly two months lying upon his back, suffering under extensive gangrene, as the result of extravasation of urine."—*London Lancet.*

